# Extra-Limites

**Published:** 1827-07

**Authors:** 


### EXTRA-LIMITES.

### PRIZE HOSPITAL REPORT, *No.* II.

MR. GEORGE BURY, PUPIL,

WINCHESTER COUNTY HOSPITAL, HANTS.

### I.

### INTERMITTENT FEVER.

THE prevalence of ague both in the city and neighbourhood of Winchester, during the present season, has been beyond precedent. I may state that of the number of in and out-door patients, admitted weekly into the County Hospital within the last three months, on the average one-third have been subjects of intermittent fever. The disease is met with in all parts around, and all situations, and does not appear to be more frequent in one place than another. The cause of the increased frequency of this complaint seems obscure, but I think it may be fairly attributed, in great measure, to the excessive heat of the summer and autumn of the past year, having been productive of augmented decomposition of vegetable matters, and a consequent increase of evaporation.

In the cure of ague the cinchona and quinine are in general use in the practice of the Hospital, and have been found universally successful, either with or without the additional employment of emetics, purgatives, opium, &c. The preference is most frequently given to the quinine, on account of the efficiency of small doses of the salt, and the advantages, in consequence, over the comparatively large quantities of Peruvian bark usually required; and within my own observation, the bark given in substance, though equally certain in its result, takes a greater length of time in subduing the disease, than when administered in the form of sulphate of quinine. Illustrative of the speedy effects of which, the few following cases are briefly subjoined.

*Cases.*

*Case* 1. Jesse Scorey, 65 years of age, was admitted into the hospital on the 18th of April, under the care of Dr. Phillips, having ague of tertian type, which had existed for twelve days. The paroxysms were severe, and lasted about ten hours; they came on at first three hours earlier after each intermission, but the two last have succeeded each other regularly on alternate nights, at 12 o'clock. The patient is uneasy during the intervals, complaining of difficulty of breathing, and pain in the head. He expects the cold fit to return at the usual hour to-night. Three grains of the sulphate of quinine were ordered to be taken every sixth hour in the form of pill.

*April* 19*th.* The man took two pills before the approach of the expected paroxysm, which was less severe, and continued five hours only. P.

21*st.* The ague did not return at the usual and expected time last night. P.

25*th.* He persisted in taking the quinine up to the present date, when he was dismissed, having had no return of the complaint since the day of his admission.

*Case* 2. George Sharpe, ætat. 15, was admitted, April 25th, under the care of Dr. Littlehales. He states that, fifteen days ago, he was attacked with ague, which returned at such irregular periods that he could not be at all certain of its approach: latterly, however, the attack has come on every other morning, at 8

o'clock. Ordered a purge of calomel and jalap immediately, and thirty drops of laudanum to be taken half an hour before the approach of the ague.

*April* 26*th*. Upon inquiry we learn that the paroxysm returned at the usual hour this morning, and that the different stages of the fever were of comparatively short duration, though not much mitigated in intensity. Rep. haust. anod. adveniente rigore.

3 o'clock, P.M. He complained that the well-known sensations preceding a paroxysm were now present; a repeated dose of laudanum was given, which in a few minutes seemed to check all unpleasant feeling, and entirely prevented the attack.

28*th*. The paroxysm returned, rather before the usual time; but was considerably shortened by previously taking the tinct. opii, as ordered before. R. Quin. sulph. gr. iij. 6tâ. quâque horâ sum.

30*th*. The ague did not return. Continue the quinine.

*May* 2*d*. No return of the ague since last report, and he was this day discharged.

*Case* 3. John Eldridge, æt. 31. Admitted under Dr. Phillips, May 9th. States that he was first taken about six weeks since. That the ague has been twice subdued by taking a mixture (in which bark was an ingredient) twice a day in brandy, and has returned each time after an interval of fourteen days. The fever makes its approach every other day, but at uncertain hours. It lasts but little more than one hour. R. Pil. quin. sulph. 6tis horis sumend.

*May* 10*th*. Ague came on this morning, and was of half an hour's duration. P.

12*th*. Fit did not arrive at the time expected. P.

16*th*. Ague has not returned since the 10*th* inst. and the man now left the hospital.

*Case* 4. John Warner, æt. 22. Admitted May 9th under Dr. Phillips, having ague of tertian type, which first came on about three weeks ago. The fit has occurred to-day, and continued for one hour, which is the usual length of its duration. R. Pil. quin. sulph. 6tis horis.

*May* 11*th*. Ague has not returned at the expected time. P.

16*th*. There has been no return of the complaint since the day of the patient's admission. He was now discharged.

*Case* 5. Stephen Scorey, æt. 14, admitted under Dr. P. with quotidian ague on the 9th of May. The paroxysm comes on regularly at 7 o'clock, A.M. and lasts nearly throughout the day. He has been ill for a fortnight, and is very much debilitated. R. Pil. quin. sulph. 6tis horis.

*May* 10*th*. The fever appeared this morning at the usual hour, but was considerably less severe, and lasted two hours only. P.

11*th*. Ague did not return. P.

23*d*. He has experienced no return of the ague; but was retained in the hospital up to this date, taking the quinine latterly twice a day to restore his strength.

### II.

### AMPUTATION.

Of capital operations performed at this hospital, amputation of the extremities is the most frequent. The circular incision, when practicable, is generally preferred, though cases sometimes occur in which the flap-operation is required. Linen retractors are on most occasions used in amputations of the fore-arm and leg, but seldom in those of the arm and thigh. The fore-arm is usually amputated at a short distance from the elbow; and when amputation is necessary for disease or injury of the hand, the wrist being in a sound state, it is performed between the extremities of the radius and ulna and the carpal bones, in preference to amputating higher up, and dividing the annular ligament; thus destroying the connexions of the different tendons, the consequences of which are the burrowing of purulent matter between the muscles, and the formation of sinuses extremely difficult to heal. The arm, leg, and thigh, are at all times removed at the places most generally recommended; viz. about three inches above the olecranon; between four and five above the patella; and four inches below the patella. The divided arteries are secured by dentist's silk, waxed, and, when applied on the larger vessels, for the most part doubled. One of the ends of the silk is cut off a little beyond the knot. Rollers are always used to give support to the parts, as they have been here found, upon experiment, to be attended with decidedly good effects. The integuments are preserved in contact by strips of empl. resinæ, and when a flap is made, sutures are employed also. Some simple cerate spread on lint is laid over the straps; compresses of their linen are then applied, and kept in situ by a light bandage.

The result of the amputations performed in Winchester may be learned from the brief statement of the principal of those that had taken place during the last two years, which I here insert without comment.

| | No. | Cured. | Died. |
|---|---|---|---|
| Amput. at shoulder joint | 1 | 1 | 0 |
| Do. of arm . . . . . | 3 | 2 | 1 |
| fore-arm . . . | 1 | 1 | 0 |
| at wrist joint . . | 1 | 0 | 1 |
| of thigh . . . . | 10 | 7 | 3 |
| leg . . . . . . | 7 | 6 | 1 |
| through metat. bones | 2 | 2 | 0 |
| Total | 25 | 19 | 6 |

The subsequent cases requiring amputation have come under our notice at the Winchester Hospital, during the present quarter.

*Case* 1.—*Amputation of the Thigh.*

Anne Jupe, ætatis 14, of scrofulous diathesis, was admitted under the care of Mr. C. Mayo, on the 1st of March, to undergo amputation in consequence of disease in the right knee. She had been an in-patient of this Institution for a considerable time during the latter end of 1826, for the relief of the same complaint, when, after numerous remedies had been employed, she left at a time the disease was in a more quiescent state, but the joint was incapable of motion, neither could she rest the limb firmly upon the ground. During the interval from her quitting the hospital to her re-admission, the joint remained enlarged and painful, and she could not walk without suffering an increase of pain. The incurable nature of the disease being pointed out to her, amputation was proposed, to which she consented, and the limb was removed by the circular incision on the 5th of March. The bleeding vessels were secured by ligatures; the integuments were brought together, and adhesive straps applied from below upwards, with compresses and bandages in the usual manner.

The girl experienced great relief from parting with the diseased member. The stump was dressed on the fifth day, (which is the usual period of removing the dressings after amputation at this hospital,) and was found to have united favourably. Some time elapsed before the ligatures came away. The patient gradually recovered from the effects of the operation, and left the hospital cured, the 18th of April.

Upon examination of the knee-joint, the synovial membrane was discovered to be considerably thickened, and lined internally by a solid grumous substance; the cartilages of the condyles of the os femoris, and head of tibia, were partially absorbed, and pus was contained in the cavity of the joint.

*Case* 2.—*Amputation of the Thigh.*

Mary Taylor, æt. 40, was received into the hospital, Nov. 22d, 1826, labouring under rheumatism, and was placed under the care of the physician. The rheumatic pains were relieved by the means usually employed; but, towards the latter end of January, 1827, acute inflammation took place in the synovial membrane of the left knee, for which she was attended by Mr. H. Lyford. The constitutional disturbance was great; the joint was immensely swollen; and the pain in it excessive, but was very much alleviated by the constant application of a lotion made with extract of belladonna, spirit of wine, camphor mixture, and water. The sympathetic fever was considerable and lasted long; the strength was supported by bark, wine, and other stimulants; the knee-joint was rested on pillows, with the limb flexed, and the above lotion constantly applied.

*March* 5*th*. The fever having in a great measure subsided, and as it was considered that the patient's constitution would sink under the irritation of the diseased joint, the limb was this day amputated by the circular incision. The arteries and *femoral vein** were taken up and secured by ligatures, and the stump was

* It is customary with the surgeons of this hospital to secure the bleeding veins, a practice which they have never known to be attended with unpleasant effects.

dressed in the usual manner, the integuments being brought together from below upwards. The wound united by adhesion, but a most profuse discharge was kept up in the neighbourhood of the ligatures. The strength of the patient was maintained by the copious administration of wine, porter, sulphate of quinine, &c. About the fifth week the stump was poulticed in consequence of the quantity of the discharge being undiminished; one or two ligatures only had been cast off, and the stump was exquisitely painful and tender. The remaining ligatures separated soon after the application of the poultices, which were continued to the end of April, at which time the purulent discharge had nearly ceased, and the sinus formed along the course of the ligatures was healed.

No examination of the diseased joint was made in the presence of the pupils at the hospital.

*Case* 3.—*Amputation of the Leg.*

W. Batchelor, æt. 37, was admitted into hospital, March 29th, in consequence of disease in the right ancle-joint. There was great enlargement of the parts surrounding the joint, and several sinuses, which gave issue to an abundant discharge of unhealthy matter, were connected with it. On examination with a probe, diseased bone was felt at the extremity of each sinus. The patient's sufferings were and had been extreme, and the constitutional disturbance was considerable. It was Mr. Lyford's opinion that sloughing of the tendons had taken place, and that the disease of the bones of the ancle was such as to preclude the possibility of saving the limb. The man's health was attended to, and poultices with fomentations ordered to be applied twice a day.

*April 9th.* The leg was removed by the circular incision at the usual place below knee. Mr. Lyford used a large catling, which he found more expeditious than the common amputating knife. Six or seven arteries required ligatures. The integuments were approximated from side to side, and the dressings applied after the usual manner. The stump was dressed on the sixth day, and most perfect adhesion was found to have taken place.. But the patient, whose constitution was broken up by previous habits of intemperance, never rallied, and the stump and knee, after the few first dressings, began to assume a sloughy aspect. Poultices were used, and wine, bark, with other stimulants, liberally administered, but he gradually sunk, and expired on the evening of the 9th of May.

The body was not examined.

*Case* 4.—*Amputation of the Thigh; the only ligature employed remaining on the vessel a long time after the stump was healed.*

Elizabeth Hathaway, nine years of age, came into the hospital, March 7th, under the care of Mr. W. Wickham, for the purpose of having her right leg removed, in consequence of an affection of the knee-joint. Numberless remedies had been previously tried, both in this hospital and in private, to arrest the progress of the disease, but it gradually advanced during several months, undermining the poor girl's health. The limb was removed by amputation on the twelfth of March. The femoral artery alone required to be secured; for which purpose a waxed ligature of strong silk twisted double was employed. One end of the ligature was cut close to the noose, and the other left hanging out at the lowermost part of the stump. The integuments were brought accurately together from side to side, and retained in contact by adhesive straps, compress, and bandage, applied as usual. The stump was again dressed on the fifth day, and found to have united most favourably; the line of union was about one-sixth of an inch in breadth, excepting at its two extremities, from one of which the ligature depended. Strips of plaster were re-applied, interposing two longitudinal pledgets of lint between them and the stump on either side of the line of union. The dressings were renewed every second day up to the 26th of March, at which time the stump was perfectly healed, save the end containing the ligature, and the other, which was touched with the lunar caustic, and lightly covered with a small piece of lint. A considerable quantity of good pus daily escaped from the vicinity of the ligature till within a few days of its separation, which did not take place before the 12th of April, when it was followed by a flow of matter, apparently contained in a cyst formed round its extremity. A small quantum of purulent fluid as well as serous, which had burrowed in the course of the ligature, was for a few days squeezed out through the orifice left by the latter. The sinus, however, soon spontaneously closed, and every part of the stump was perfectly cicatrized by April 18th.

*Remarks.*—In this case it will be observed that the stump was healed in the short space of a fortnight, but that the patient could not avail herself of the advantages of so speedy an adhesion of the integuments, because the ligature still remained on the artery. Consequently, she was confined to her bed for three weeks after the cicatrization of nearly the whole stump, as any exertion on her part might, at any period before the separation of the ligature, induce dangerous hæmorrhage. I think it not improbable that the irritation, in comparison trifling, caused by the existence of only *one* silk, was particularly favourable to so quick an union of the parts; since it has been satisfactorily ascertained by Mr. Lawrence and others, that all large wounds, in which there is need of ligatures, would unite more speedily, if the necessity of these extraneous bodies could be superseded. Every one, accustomed to the dressing of stumps after amputation, must have frequently witnessed the uncertain length of time before ulceration of the coats of the vessel takes place, to allow of the removal of the ligature. In the present case the ligature was retained a long time, though not unusually long, for in another case of amputation of the thigh (Case 2), in an adjoining ward of this hospital, all the ligatures were not cast off till the 20th of April, although the operation was performed a week prior to the one which is now under consideration. Mr. Wickham, regretting the unavoidable necessity of the patient's confinement to bed for so long a period after the healing of the stump, and knowing the success of Mr. Fielding, of Hull, in the employment of ligatures made of silk-worm gut, determined on making use of them, in the manner recommended by that gentleman, in the following case:

*Case 5.—Amputation of the Thigh. Ligatures of Silk-worm Gut applied on the Vessels.*

George Staples, ætat. 9, in appearance delicate, but having always enjoyed perfect health, was made an in-patient of Winchester Hospital, on the 14th of March, having extensive disease of the right tibia, commencing from near its head, and continuing to the ancle-joint. The integuments of the anterior part of the leg were discoloured, and perforated by numerous sinuses communicating with the shaft of the bone, and two or three on either side of the ancle, from which a copious discharge escaped. The patient stated that the leg had not been affected for more than six weeks; but from the existence of diseased bone to such an extent, his testimony can scarcely be relied on. It was not painful, except when touched, or handled roughly. Fomentations and poultices were applied, the action of the bowels regulated, and, as the boy's health was declining, and the disease considered irremediable by any other means, amputation was performed above the knee by the circular incision on the 6th of April, Mr. Wickham, thinking it impracticable to amputate below, because the head of the tibia was enlarged, and (as it afterwards proved to be) very probably carious. The femoral artery, four others, and one small vein which poured forth blood freely, were secured with silk-worm gut made ductile by previous maceration in warm water, as recommended and practised by Mr. Fielding; both ends of the ligatures were cut close to the vessels; the integuments were brought into contact by adhesive plaster from side to side; the stump dressed in the usual way, and the patient was placed in bed, having the tourniquet loosely applied round the thigh by the direction of Mr. Wickham, as a reserve in case hæmorrhage might ensue. The boy bore the operation ill, but was calm and tranquil up to the fifth day, when the superficial dressings were removed, and the adhesive straps snipped with the scissors over the surface of the stump, to give outlet to any matter that might have accumulated beneath them, but none escaped; some simple cerate spread on lint was then laid upon the strapping, with a lightly applied compress and bandage. All the dressings were removed on the seventh day. The stump had united by adhesion, though the upper edges of the integuments were some distance from each other; it was dressed in the same manner as the case preceding. On the evening of the day after dressing (April 13th), owing to the restlessness of the patient, the bandage slipped forward, in consequence of which the dressings were again applied, rather prematurely, but fortunately no adhesions of the integuments were materially disturbed. The patient was free from any pain in the stump, and had on that day a little roast meat for dinner.

*April 21st.* The bandage and dressings have again become loose from the boy's turning in bed last night, and the integuments, being deprived of their wonted support, have retracted, and the wound of the stump is consequently enlarged.

*May 4th.* The wound has been dressed with regularity alternate days, and is now completely cicatrized. The quantity of discharge at each dressing has been inconsiderable, no more than ordinarily issues from a granulating surface of the same dimensions situated in other parts of the body. No pain or tenderness is discoverable by pressing near the extremity of the stump; which affords good reason to believe, that the small portion of gut used in the ligatures has been taken up by absorption. The little patient has sat up the greater part of the day during the last week.

*16th.* The boy was retained in the hospital up to this time, by desire of Mr. Wickham, in order that further progress of time might confirm the fact of absorption of the ligatures.

*Remarks.*—That the silk-worm gut ligature can be employed with safety, the preceding case tends to prove; and as far as an isolated instance has weight, in addition to the cases given by Mr. Fielding, we may infer that the silk-worm gut may be applied on vessels with greater advantage than any other substance that has hitherto been proposed. In perusing the above case, it cannot but strike the reader that the cicatrization of the stump was considerably retarded by the displacement of the dressings, owing to the restless motions of the patient; had not this circumstance occurred, it is Mr. Wickham's opinion that it would have been effected in little more than a fortnight. But, independent of this, the wound was healed in the present case in less time than the one preceding by nine days, although the adhesion was by no means so favourable; and the boy was able to sit out of bed a week before it was healed, a privilege which Hathaway, though otherwise fully equal to it, was prevented from enjoying until the ligature separated.

The number of vessels tied in the present case serves not a little to shew, that hæmorrhage can be effectually arrested by the application of silk-worm gut; and as we have no grounds for disbelieving that the particles of gut have been acted upon by the absorbents, for the same reason the case points out to us, that this animal substance is capable of being absorbed in considerable quantity even in a young and delicate constitution. Future trial and experience will decide on the superiority or inferiority of the silk-worm gut ligatures; but Mr. Wickham is so impressed with the belief of the former by the issue of the above case, that he has resolved upon using them again the first opportunity that presents itself in his hospital practice; and I had indulged the hope that, before the termination of this report, I should have been enabled to have given the result of some other case or cases similarly treated.

### III.

### DISEASES OF THE HIP-JOINT.

*Case 1.—Chronic Inflammation of the Synovial Membrane.*

Treated by W. J. WICKHAM, Esq.

SARAH BULPITT, 22 years of age, was admitted into hospital on the 4th of March, on account of an affection of the left hip. She states that she has experienced in walking some pain accompanied with limping for nearly two years. She complains of pain rather severe confined to the joint, which is swollen externally, and tender to the touch, particularly in the groin. No elongation or abbreviation of the limb can be discovered on examination; nor has there been either at any former period. The thigh and leg have retained their natural size. Her health is undisturbed; appetite good, and she sleeps tolerably well at night. She was desired to keep her bed, some opening medicine was ordered, and blood was taken from the joint by cupping to ℥x. House Diet.

*March 6th.* No sensible relief has been derived from the cupping. A seton was to day introduced behind the trochanter major.

*10th.* Pain of the hip-joint is diminished by the counter-irritation of the seton, which discharges plentifully.

*21st.* Patient has, till within the last few days, been surprizingly easy, but she now complains of an increase of pain in the hip, which disturbs her rest by night; the secretion of the seton is less in quantity. Mr. Wickham ordered the application of a blister near the seton, and one grain of opium to be taken every night.

*27th.* Considerable benefit has attended the use of the blister, which is now healed; a poultice was directed to be applied over the seton, to encourage the discharge of matter.

*April 4th.* She continues easy; sleep is still procured by the opium taken at night.

*12th.* The discharge from the seton has increased since the cataplasms have been employed; the tenderness and swelling of the joint are relieved by the seton. The blister was to-day repeated in consequence of a slight return of pain.

*21st.* The joint has been easy, subsequently to the application of the blister: the observance of rest is still enforced, and the seton-threads are continued. The bowels have been obstinately constipated, but have, at length, yielded to repeated calomel purges. Another blister was ordered to be applied to the hip, and to be dressed with the Sabine ointment.

*27th.* The patient complains of occasional pains in the hip, but not of a severe nature.

*May 2d.* Hip easy; the seton has nearly ulcerated through the skin.

*5th.* The seton-threads separated on the 3d, and the patient has been since in great pain, both in the hip and shoulder likewise, which she says was affected before her admission, but has been altogether free from pain, since the remedies mentioned above have been applied to the hip. A blister was now ordered for the hip, and shoulder also.

*12th.* Pain of the hip and shoulder-joints has been successfully relieved by the blisters. The woman still keeps her bed, and no motion is allowed. The blister to the hip was this day repeated.

*19th.* Patient sits out of bed the latter part of the day, and says the hip is only painful at times. She has had recourse to another blister, which she used as soon as the former one was healed.

*24th.* She has complained of severe pain in the knee, and a little above that joint, but it has been greatly relieved by applying a blister last night immediately above the patella.

*27th.* The patient has at present a tolerable use of the limb; she can walk without suffering pain, but complains of slight swelling of the integuments of the knee-joint and calf of the leg. There is no alteration from the natural form and appearance visible in the hip; nor any pain, as a plentiful discharge comes from the wound made by the seton, which she has always found more effectual in mitigating pain, in proportion to the quantity of discharge secreted. Her health, which, it will be seen in the report, has undergone no change, is not in the least affected; and, from the present features of the case, we may infer that, by perseverance in the plan of blistering, the local disease will be, ere long, removed.

*Case 2.—Apparent Elongation of the Thigh, with Wasting of the Nates.*

Treated by W. J. Wickham, Esq.

Richard White, æt. 18, was admitted, February 21st, with disease of the right hip-joint. He complained of pain proceeding from the joint down the anterior and inner part of the thigh to the knee, where it was most severe. *Pain* in the thigh had been constant for the last three months, but not in the same situation as at the above date. He had been an In-patient of the hospital in the early part of January last, under the care of one of the physicians, at which time the pain extended from just below the trochanter major down the *outer* side of the thigh, to the outer malleolus. On the supposition that the disease was sciatica, he was then treated accordingly, and the operation of acupuncture was twice performed. But the pain still continued, and the boy referred it at different times to different parts of the thigh: from which circumstances, and the patient's coming from a parish workhouse, together with some others, the symptoms not being sufficiently well-marked as indicative of disease of the hip, it was thought that his complaints were counterfeited, and he was shortly dismissed the house.

On his re-admission under Mr. Wickham, at the time above-mentioned, about ℥viij. of blood were abstracted from the hip by cupping, after which operation the pain was diminished. When the joint was examined, on the following day, no particular enlargement or swelling was observable; but there was exquisite tenderness in the groin, with pain produced by pressure; the nates of the right side were wasted, and on bringing the two lower extremities together in a straight line with the body, the patella of the right knee was full two inches lower than the other. The easiest position in which the patient could lie was on his back, inclining rather to the side affected, with the body bent forwards and downwards. A seton was inserted in the groin, by direction of Mr. Wickham. The boy was ordered to be kept perfectly quiet in bed. His bowels were evacuated by house medicine. He had no fever, and, in other respects, he might be said to have been in good health. House diet.

*March* 1*st*. Almost immediately after the insertion of the seton, the pain in the vicinity of the hip was surprizingly diminished, as was also that below in a considerable degree. The discharge is copious, and the counter-irritant is not productive of much pain. Tenderness in the groin less.

10*th*. The patient complains of an increase of pain both in the hip and knee. The discharge from the seton is becoming less by degrees; a light linseed-meal poultice was ordered to be applied over it, to promote the secretion of pus.

15*th*. Pain in the hip and knee-joints still greater; discharge from seton increased. A blister is to-day directed to be applied behind the greater trochanter.

21*st*. The pain in the above joints was considerably alleviated by the blister for the first few days subsequent to its application; but the pain in the knee has returned with greater violence than before, on which account a blister was again applied immediately above it anteriorly.

26*th*. The last blister has removed the pain from the knee; a plentiful discharge from the seton has been promoted by the poultices, which are now ordered to be discontinued. The patient has hitherto observed the most absolute quietude, and he says he is free from any pain in the joint or limb.

*April* 1*st*. Patient complains occasionally of pain in the hip and loins; the seton still discharges, though but sparingly; the threads will apparently soon drop out from the ulceration of the integument. Tenderness of the groin has been long removed.

7*th*. The seton-threads to-day came away. The boy is quite easy.

13*th*. The whole extremity is quite free from pain, and can allow of rotation either inwards or outwards; but the thigh is still as much longer than the other as at any former period. The wound left by the seton is nearly healed, and dressed with some simple cerate.

19*th*. The patient still keeps his bed, and complains of occasional pain in the outside of the thigh.

21*st*. No pain or tenderness near the hip, but grasping the thigh, a little above the knee, with the hand, causes pain. Mr. Wickham thought the elongation of the thigh rather less. Another seton was now passed behind the great trochanter.

24*th*. The boy thinks the pain in the vicinity of the knee is diminished; but, by pressing the head of the femur against the acetabulum, there is pain excited in the knee, which proceeds about half way up the anterior part of the thigh.

30*th*. The lad is easy, with the exception of pain in the knee, where a blister was directed to be applied.

*May* 6*th*. The pain in the knee has been relieved by the blister. He still continues tranquil: but no alteration of importance can be observed in the length of the right limb.

15*th*. The boy has latterly experienced pains, of rather a severe description, down the outer part of the thigh to the knee. An abundant discharge of pus is maintained by the seton behind the trochanter major.

22*d*. The pains in the outer side of the thigh are less frequent; there is no pain in the hip.

27*th*. Patient suffers but little. The lengthened state of the thigh is unaltered; indeed, he himself thinks it is increased. The wasting of the nates has not been augmented since he has been in the hospital, a period better than three months; and the muscles of the thigh and leg have fallen away from disuse only. He has been rigorously confined to bed; and so, I fear, he must continue some time longer, until the diseased action of the joint be arrested by the counter-irritation of setons and blisters; and, even then, the probability is, that the affected limb will be permanently longer than the other. His health has never been disturbed, and, from his youth, he will be enabled to go through the future confinement and necessary treatment.

*Case* 3.—*Shortening of the Thigh.*

Treated by C. Mayo, Esq.

Isaac Gustar, æt. 23, and of short stature, was admitted into hospital on the 11th of April, on account of an affection of the left hip, of about nine weeks' standing. He complains of severe pain, confined to the hip, and feels the joint, as he expresses it, "stiff." The nates and whole extremity of the left side are wasted; the limb about two inches shorter than the other, and the least exertion of the leg, or pressure near the seat of disease, is productive of pain. The patient's strength is much reduced; he labours under sympathetic fever to a considerable degree, and profuse nocturnal perspirations, and is a stranger to the beneficial effects of sleep. Pulse is quick and feeble, 112: tongue coated, and appetite very defective.

The day following his admission, blood was abstracted from the neighbourhood of the hip by cupping, which, in some measure, procured relief. Equal parts of Dover's powder and extr. conii, made up in pills, were taken at night.

*April* 16*th*. The man has gone once into the hip-bath, and had leeches applied to the joint, which treatment has been serviceable. A caustic issue was now made over the great trochanter; and the next day the pain was considerably less, as well as the stiffness complained of. A poultice was applied to the slough.

19*th*. The pain in the groin is excessive, and the man has passed a sleepless night. The poultice to be continued until the separation of the slough takes place, and the dose of the pills to be increased.

25*th*. The pain has gradually diminished, and the patient is at present easy. The slough was to-day removed, and peas were laid on the surface of the eschar in the ordinary way. A tonic mixture of bark and acid has been prescribed, and the sedative pills are continued.

29*th*. Since the last date, patient has been tolerably free from pain; but no alteration can be discovered in the hip-joint, or the length of thigh. He continues the mixture and pills, and remains out of bed a short time in the course of the day.

*May* 4*th*. The man is easy, and feels stronger. Pills have been omitted. Contin. mist.

10*th*. Hip continues free from pain, but he complains of an increase of stiffness therein. A plentiful discharge of matter has been kept up by the issue: and his sleep at night is refreshing. His countenance is materially improved. A pint of porter is allowed him daily. Cont. cinch. c. acid. dil.

18*th*. Patient seems improving daily; he makes no complaint. Hip is easy, but no alteration has taken place with respect to the length of the extremity. The porter and bark mixture are continued.

23*d*. He reports he is gaining strength by degrees; he suffers no pain. Cont.

27*th*. The man is gradually recovering his strength, and the hip-joint continues free from pain. He can endure slight motion of the limb, without experiencing any ill consequences. The thigh is still shortened, and but slight alteration has occurred in the whole limb since his admission into the house; but, under the employment of the issue, and the liberal exhibition of tonics, with the advantage of his age, an unavoidably imperfect use of the limb may be expected, though the restoration to its proper length is doubtful.

*Case* 4.—*Shortening of the Thigh.*

Treated by W. J. Wickham, Esq.

Eliz. Spencer, æt. 20, of weak constitution, was admitted into the hospital, April 4th, on account of a disease of the left hip-joint, which had been of eighteen weeks' standing, but for which no medical aid had been employed. Upon examination, the left extremity was found to be considerably shorter than the opposite one; the knee was turned inwards; there was extensive wasting of the glutæi muscles, and the femur was drawn upwards by their antagonists; anterior to, and a little below, the superior anterior spinous process of the ilium, was a large swelling, slightly red, which was extremely painful on pressure, but no glands in the groin were enlarged; the trochanter major formed a tumour nearly globular at the upper and back part of the hip-joint. The patient could lie in the following position only with ease, viz. on the right side, with the head and shoulders bent forwards and downwards toward the trunk, keeping the affected joint upwards. She laboured under severe hectic fever, and was deprived of sleep at night, though the appetite was good, tongue clean, and alvine evacuations natural. A peculiar glassy appearance of the lucid cornea was visible. Some diaphoretic medicines were ordered, and a light nutritious diet allowed; a seton was cut near the seat of pain, and, on the following day, the patient said she spent the night almost free from pain.

*April* 12*th*. The pain of the hip is surprisingly diminished, tenderness on pressure less, and the febrile symptoms have greatly subsided. A good discharge has been kept up by the seton. Cont.

16*th*. Hip tolerably easy, but the woman complains of a slight diarrhœa and pain of the abdomen. Ordered the saline mixture with six minims of laudanum every five hours.

20*th*. Looseness of the bowels checked; patient is at present easy, and thinks she is somewhat stronger; hip free from pain of any importance, and a copious secretion comes from the seton. The saline draughts to be continued without the opium.

25*th*. Diarrhœa has returned; the hip is now uneasy, and the joint and thigh appear more wasted: the seton threads keep up a plentiful discharge. Ordered pulv. cretæ comp. c. opio, ℈i. post liquidas sedes sing. sumend. Cont. haust. salin.

29*th*. The diarrhœa continues unabated, and the patient is considerably weaker; the appetite is very indifferent, but the pain of the joint does not prevent her sleep. Continue. Dieta ad libitum.

*May* 4*th*. Hip has been painful; but is now easier; a poultice of linseed has been applied to promote the discharge from the seton, and in consequence of a swelling, which appears to contain matter, situated a little above the wound made by the seton-needle. Bowels not so relaxed. Pergat. medic.

10*th*. There is a copious discharge of pus in the poultices from an opening in the swelling mentioned above. The joint is tolerably easy, and the action of the bowels more regular. Patient is rather more feverish, and perspiration increased. Her sleep is not much disturbed. Contin. mist. salin. et omitt. pulveres.

15*th*. There is no visible alteration in the girl's health, if any, rather for the worse. The hip-joint is not painful; she sleeps better at night, but does not awake refreshed; she takes but little nutriment; bowels are irregular; pulse quick, and weak. Poultices are continued, and an abundant discharge issues from the opening, and the seton also. R. Quin. sulph. gr. iij. bis quotidie sum.

20*th*. Patient seems weaker, but makes no complaint; the discharge from the opening is somewhat less in quantity. Appetite has improved a little since last report. The poultices are continued to the part, and a mixture of decoct. cinchonæ c. tinct. taken ter die, has been substituted for the quinine.

27*th*. Debility gradually increasing, although her appetite is not bad; sleep is procured by taking opium. There is no considerable pain in the neighbourhood of the hip; but a copious discharge of matter issues from the sore left by the opening in the groin, which is deep and not disposed to granulate. Some of the unguent. hydr. nitrico-oxyd. diluted, is ordered to be applied to the wound; tonic medicines and nourishing food are given freely; but the fatal termination of the case appears to be, by degrees, though slowly, approaching.

*Remarks*. The above instances of diseased hip-joint illustrate, by the *first*, the disease of the synovial membrane, independent of diseased cartilage; by the *three latter*, that disease which originates in the *cartilaginous surfaces* of the joints. The disease of the synovial membrane seems to produce but little alteration in the appearance of the limb in its first stages, until the cartilages become affected. But it will be found that *very early* in the disease, when the cartilages are first affected, the limb becomes altered. The *second case* illustrates the apparent elongation of the thigh (evidently arising from a depression of the pelvis on the right side, as described by Mr. Brodie) which happens in the early stage, continuing an unusual length of time, and almost amounting to a chronic elongation.

The two latter cases shew the subsequent stage of shortening of the leg.

The treatment best adapted for the synovial disease, by the practice of Mr. Wickham, appears to be the frequent application of blisters; but where the cartilages are primarily affected, setons are more decidedly useful.

---

### IV.

### SECONDARY SYMPTOMS OF SYPHILIS.

The three following cases, out of the many that have fallen under my observation at the hospital during this season, I shall here introduce, as illustrating the decisive practice of Mr. Wickham in the treatment of symptoms, occurring subsequent to the supposed cure of syphilis, usually denominated secondary; as well as those which appear after the abuse of mercury.

*Case* 1.—*Venereal Sore Throat, treated by Mercury.*

Charles Banks, æt. 24, groom, was admitted into our hospital on the 16th of March, having an ulcer of the throat, situated below the uvula. Its surface was a little larger than a sixpence, and by the patient's account gradually increasing; respiration was rendered difficult, and a considerable quantity of bloody mucus was expectorated from it. The man denied ever having had the venereal disease in any form, or having to his own knowledge used mercury, and affirmed that he had never been salivated. He further stated that he first discovered that his throat was affected, after recovery from taking cold a few months ago. By the appearance of the ulcer Mr. Wickham was convinced of its being syphilitic, notwithstanding the patient's assertion to the contrary. His bowels were directed to be kept open by some pills composed of extr. coloc. comp. and pil. hydr. taken every night; and a detergent gargle of nitro-muriatic acid, with infusion of roses, to be used frequently, was prescribed. House diet.

*March* 24*th*. The ulcer is somewhat cleaner, but continues to spread. It occupies nearly the whole space between the tonsils; its shape is rather oblong than round, and its true syphilitic character is now well marked. Mr. W. prescribed gr. x. of blue pill to be taken night and morning, and ordered the gargle to be continued.

30*th*. The mercury has produced severe purging with tormina, wherefore ¼ of a grain of pulv. opii was added to every five grains of the pill mass. The appearance of the sore is unaltered, but the secretions are increased by the action of the mercurial. After taking the blue pill conjoined with opium for two days only, the diarrhœa and griping returned, and he was obliged to intermit the mercury for a few days. It, however, was resumed on the 5th of April, and he continued taking it united with opium until the 17th, when the mouth was affected, and there was manifest alteration in the ulcerated surface. The centre of the sore was at that time clean and granulating, his breathing was less laborious, and not so stertorous at night. The dose of the pill was then also diminished to ten grains in the evening.

*April* 21*st*. The ulcer in the throat has healed, but the man complains of obstructions in the nose. Five grains of blue pill are directed to be taken at night only.

26*th*. The mercurial pill was to-day discontinued.

*May* 1*st*. Nasal obstruction the same, but no denuded bone can be detected in either nostril. A muriatic-acid injection was ordered to be used occasionally.

9*th*. The man left the hospital as cured, though the impediment in the nasal organs continues. In all probability exfoliation of bone will take place from the nose at no very distant period.

Mr. Wickham, though fully satisfied that the sore throat was venereal, postponed the exhibition of mercury, in order to try the effect of aperients, and the local remedy, as the absence of primary symptoms of syphilis was declared by the patient, and no marks thereof were visible on the genitals. How long the disease had existed in the constitution, cannot be determined by conjecture: but it is probable that the original affection was contracted at the distance of some years, and that this circumstance gave colour to the man's firm denial. The case shews us the extreme caution requisite in placing credit on the historia morbi given by the patients themselves, and that the mode of treatment should be guided by the judgment and discrimination of the practitioner.

*Case* 2.—*Syphilitic Sore Throat, together with Inflammation of the Eyes, treated by Mercury.*

John Smith, ætatis 32, admitted April 25th, in consequence of ulcers of the throat, which he first discovered about two months ago. Two of them are situated near the right tonsil, one immediately above the uvula, and a fourth near the left tonsil. They are small in size, and not deep; their base is slightly yellow; edges even, but irregular; and the surrounding parts are inflamed. In both eyes there is a considerable degree of inflammation, both superficial and deep-seated, with irregularity of pupil, and deposition of lymph. The eyes have been affected a few days only. He was feverish, and had a loaded tongue. He states that about three years from the present time venereal sores appeared on the penis, for which he was taken into one of the hospitals at Bristol; there it appears that mercury was given in alterative doses, and discontinued as soon as the sores healed, and the mouth became gently affected. He positively asserts that he has not since received any fresh venereal infection. His bowels were ordered to be purged with house medicine, and a muriatic acid gargle was prescribed.

*April* 30*th*. Inflammation of the eyes greatly abated, and sight more perfect: but the ulcers in the throat, though rendered cleaner by the use of the gargle, have spread downwards, and are larger than at the period of his admission. The gargle was directed to be continued, and the following exhibited; R. Hydr. submur. gr. ij. pulv. opii. gr. ss. ℳ mané et vesp. sumend.

*May* 1*st*. The eyes were attacked with a return of inflammation, similar to the former attack, but more severe. Four leeches were applied to each temple. Calomel and opium to be continued.

2*d*. The inflammatory action of the conjunctiva, and also that which has taken place internally, are much reduced, but the sight is dim. The sores in the throat are cleaner, and the surrounding copper-coloured inflammation has disappeared. Gums swollen and tender. Pergat.

4*th*. Mouth affected. Ulcers clean and disposed to heal. The eyes have recovered their natural appearance, but the dimness of sight still exists. Calomel and opium continued.

6*th*. Secretion of saliva copious. Ulcers healing. Vision still imperfect. The calomel and opium to be taken once a day.

8*th*. Salivation has been restrained by an alum gargle. Throat healed. Calomel and opium are omitted.

12*th*. There remains a slight degree of irregularity of the right pupil, but no lymph is left unabsorbed. Sight has not improved since last account.

16*th*. Patient was this day discharged from the hospital, the pupil having recovered its natural shape, and vision being distinct.

Mr. Wickham considered that the syphilitic disease in this case might probably have been suspended, during the three years from the patient's undergoing an alterative course of mercury, up to the period of his being received into this hospital; though he could not place absolute reliance on the evidence of the man, that no recent infection had been communicated. In either case he thought there was but one course to pursue, as he believes, from the results of his own experience, that alterative doses of mercury are inefficient in the treatment of either the primary or secondary symptoms of syphilis.

*Case* 3.—*Disease of the Bones of the Nose, occurring after the excessive Use of Mercury.*

Richard Dance, æt. 24, was admitted into the hospital, on the 5th of April, with disease of the bones of the nose. He stated that in March, 1826, there appeared sores on the penis after connexion, for which he took mercury in the form of pill, and employed it externally *during ten weeks*, and was kept under profuse salivation. The sores healed some considerable time before the mercurial action was restrained. He likewise informed us that, in the month of August last, inflammation of the right elbow-joint took place, attended with extensive tumefaction both above and below, which was shortly dispersed by cupping and leeches; and that about six weeks from that time, in the beginning of the ensuing October, he experienced violent pains in the right shoulder resembling those of rheumatism; for which, from previous history supposed to be syphilitic, he was again salivated, after having taken mercury for *six weeks*. The pain of the shoulder-joint, he said, was removed by the exhibition of the mercury, but from exposure to nightly wet and cold in November, having scarcely recovered from the effects of the last mercurial course, he was laid up for weeks. On his recovery, (which did not happen before February of the present year,) he found obstructions in the nasal passages, and incapability of breathing through his nostrils, on which account he came into the hospital at Winchester on the day above-mentioned, when the fore-going history was learned from him.

The whole bridge of the nose is now enlarged, and its integuments discoloured; pain is caused by compressing the swollen parts, and a bloody mucus is discharged in large quantity. The man says that exfoliation of bone has not yet occurred, but, by passing up a probe, denuded bone to some extent was distinguished in the left nostril. A lotion of diluted nitro-muriatic acid was ordered to be thrown up frequently during the day by a syringe, to assist the separation of dead bone; a dose of house medicine, and half a pint of decoct. sarsæ compos. per diem.—House diet.

*April* 10*th*. A copious discharge is brought away by the injection. The sarsaparilla is taken regularly, and does not disagree with the patient's stomach. Continue.

17*th*. The breadth of the nose is less, and the man can breathe through it in a slight degree. A piece of bone, the size of a sixpence, and rather thicker, to-day exfoliated. The injection and sarsaparilla continued.

23*d*. The nose is less tender on pressure, and the air-passages are more free. Another particle of bone smaller than the former one has come away from the left nostril. The acid lotion is still injected, and followed by an offensive discharge each time it is used. The decoction continued.

*May* 1*st*. Breadth of the nose is gradually diminishing, but the secretion of fetid matter has increased within the past week.

9*th*. Patient was this day discharged at his own request; several little pieces of bone having been cast off, and the nose being nearly restored to its natural form, though the fetid secretion still comes away in considerable quantity. The sarsaparilla has within the last few days produced violent head-ache, with sickness and oppression at stomach: but he has been relieved by a cathartic, saline mixture, and leeches to the temple. He was ordered to continue the injection, as it appeared probable that further exfoliation might take place.

It was Mr. Wickham's opinion, that all diseased action of the syphilitic poison was suspended in the present case; and in consonance with that belief he had recourse to the above mild and alterative treatment.

---

### V.

### DISEASES OF THE BREAST.

THE subjoined cases of diseases of the breast, requiring operations, have been lately admitted into the Winchester Hospital, and on account of the practical importance of successive reports of the diseases of that part, more than from any intrinsic value of the cases themselves, I have been induced to lay them before the reader. It will be seen that the diagnostic symptoms of Case 1 were by no means in accordance with those laid down by authors, as indicating an hydatid of the breast; nor indeed were those, by which the chronic tumour is distinguished, as distinct as usual in the second case.

Case 1.—*Encysted Tumour of the Breast.*

Eliz. Axford, æt. 35, came into this hospital in the month of February under the care of Mr. Lyford, on account of a tumour seated near the mammary gland of the left side, being the growth of about eight months. She stated that she first discovered the swelling, at the commencement of the time above specified, when the size of a filbert, and that it had progressively become larger unattended with pain. In its external appearance, the enlargement in some respects resembled scirrhus, being of the usual size of that disease, solid, and rather hard to the touch; but it had not the mobility, or circumscribed limits of scirrhous tubercle. There was no pain in the part, but the integuments were discoloured on the upper part of the tumour, and the nipple was retracted. The patient was a healthy subject, of remarkably florid complexion, and the mother of several children. The catamenia had always appeared regularly. She complained only of the inconvenience of the swelling, but she was not free from the alarm generally excited in female minds, by having a mammary disease, and she was anxious to have the breast removed. She was placed on house diet, with the occasional exhibition of aperient medicines.

*Feb. 27th.* Agreeably to the wishes of the woman, the whole breast was to-day removed; Mr. Lyford, thinking that operation necessary, as there was an unhealthy secretion from the retracted nipple. The patient being seated on a low chair, Mr. L. commenced by making two elliptical incisions from above obliquely downwards, meeting at each end, including within the ellipsis the diseased part, together with the nipple; the cellular membrane was then detached, the pectoral muscle exposed, some fibres of which were removed, and the tumour easily dissected from its connexions. Three or four vessels were secured. Two sutures, assisted by the application of adhesive straps, were employed to bring the edges of the integuments together; some simple cerate was next applied with compress and broad roller.

The operation was endured with resolution, and the woman was quite easy during the after-treatment. The dressings were removed on the fifth day; the straps of plaster were separated singly, care being taken to substitute others immediately after the removal of each. Wound united closely, the upper portion excepted. The stitches were cut away at the third dressing; and the ligatures cast off about the tenth or twelfth day. The small granulating surface above was soon healed, and the patient discharged cured on the 28th of March.

When the part removed was cut into, a cyst was divided, containing a yellow, transparent, and jelly-like substance.

### *Case 2.—Simple Chronic Tumour of the Breast.*

Susan Green, æt. 47, married, and who has menstruated regularly, was admitted on the 21st of March, under Mr. C. Mayo, with chronic tumour of the right breast, as large as a hen's egg. She first perceived the swelling, when very small, about six months previous to admission, and it has gradually increased without pain; it is somewhat superficial and moveable, and the circumscribed and indurated feel of scirrhus is wanting. Her health is undisturbed. One gland in the axilla is enlarged from the irritation of the tumour. She was ordered to take five grains of Plummer's pill at night, and to keep moistened cloths applied to the breast.

*April 6th.* Amputation of the breast was to-day performed, and the enlarged gland of the axilla removed. Mr. Mayo made his incisions obliquely downwards from right to left, on each side of the tumour. Three or four bleeding vessels were tied. The edges of the wound being approximated, adhesive plaster was applied, with pledgets of simple dressing, compress, and bandage. The dressings were renewed on the fifth day; the divided integuments adhered kindly; the ligatures soon separated, and the patient was discharged cured on the 25th.

The swelling, when cut into, appeared lobulated, consisting of larger and smaller lobes, somewhat striated, and of a yellow colour; the gland of the axilla that was removed was perfectly healthy.

There are three circumstances relating to the above case of unusual occurrence in simple chronic tumour of the mamma; viz. the advanced age of the patient; the quick growth of the swelling; and the enlargement of the axillary gland.

### *Case 3.—Scirrhous Tubercle of the Breast.*

Sarah Whitmarsh, æt. 45, was admitted into hospital on the 9th of May, having a tumour near the right mamma, which is about the size of a walnut, and which was noticed in the first place about twelve months since. It is situated on the right of the nipple, and a portion of it branches upwards; it has the usual feel of stony hardness, characteristic of malignant disease, and is attended with violent lancinating pains, occurring after short intervals, extending at present in a direction from the nipple. It is moveable beneath the integuments; the skin in front is smooth, and does not appear firmly adherent. One of the glands of the axilla is enlarged, tender, and hardened; and there is the same darting pain between it and the tumour. She does not recollect ever having had any unhealthy discharge from the nipple. The constitution of the woman is unhealthy, and is now much impaired, as she has, of late years, gone through considerable mental affliction from various causes. She is married, and has borne one living child; has been pregnant five times besides, but each time was unable to go to the full period of gestation. No alteration has yet taken place in the menstrual discharge, which is performed regularly and in proper quantity. A purgative dose of pulv. rhæi comp. was ordered to be taken immediately.

*May* 14*th*. She complains of the severe pain shooting near and through the nipple, as well as towards the clavicle. Ordered empl. ammoniaci c. hydr. to be applied to the swelling, and the following tonic to be exhibited thrice a day.—R. Inf. gent. compos. ℥x. sp. ammon. arom. ʒj. ♏

20*th*. The breast has been free from pain since the application of the plaster. Her appetite is not good, and there is some thirst; she sleeps tolerably at night. P.

27*th*. Patient's health has improved, and Mr. Lyford has it in contemplation to remove the tumour in a few days. The axillary gland has subsided.

---

VI.

CASE OF BRONCHOCELE SUCCESSFULLY TREATED BY SETON, BY H. G. LYFORD, ESQ.

THE following case assists in confirming the efficacy of the seton in the treatment of a disease, which we know from experience is sometimes attended with fatal consequences, and bids defiance to the aid of medicine.

Eliza Gibbs, æt. 13, of florid complexion and light coloured hair, and perfectly healthy, was admitted into Winchester Hospital in the autumn of 1826, under the care of Mr. Lyford, on account of an enlargement of the thyroid gland, the growth of four or five years. Various applications had been tried to effect a diminution of the tumour both by Mr. L. and his father, prior to her admission. The swelling was as large as an egg, in shape nearly round, and its circumference well defined; it felt unusually hard, and was easily moveable. Its size was gradually, but very slowly, increasing. Respiration was performed without difficulty, but deglutition was productive of pain. No arterial pulsation could be felt in the tumour. Its removal by the knife was at that time proposed by Mr. Lyford, but, at a consultation of his colleagues, it was thought expedient that the operation should be deferred until other means had been employed in the hospital. The unguent. potass. hydriodat. was ordered to be rubbed into the swelling night and morning, and the tincture of iodine was given internally.

Shortly after she commenced this treatment, Dr. Clutterbuck (who was on a visit in Winchester) saw the patient at the hospital, and recommended the application of the burnt woollen cloth, alleging that he had lately seen it employed with success in several instances.

She continued to use the ointment and take the tincture for some weeks, when she was discharged as an out-patient, the enlargement still retaining its former size and shape. Whilst an out-patient, she persisted in the employment of the same means, up to the latter end of the month of February, 1827, at which time she was again received into the house, the bronchial tumour being as large as at any former period.

1827. *March* 12*th*. No relief having been hitherto obtained by the adoption of the measures mentioned above, Mr. Lyford determined on having recourse to the seton. Accordingly he now passed a seton-needle, armed with a few threads of cotton, from above obliquely downwards through the centre of the swelling, leaving an interval of an inch and a half between the entrance and escape of the needle. No hæmorrhage or untoward circumstance impeded the introduction of the instrument employed, which was one made for the purpose, from five to six inches in length, its greatest breadth not more than ¼ of an inch, sharp-pointed, and shoulders cutting; the whole portion behind the shoulders was round, with the exception of the extremity, wherein the threads were inserted, which was slightly flattened.

A few days subsequent to the operation, the part was attacked with a trifling degree of erysipelatous inflammation, accompanied with a copious and offensive discharge from the orifices of the seton, and pain was produced by deglutition.

The inflammation happily soon subsided, and within the short space of a fortnight a reduction of the tumour was manifest.

*April* 4*th*. Discharge kept up by the seton abundant, and very fetid; the little girl has experienced a good deal of febrile excitement, which is at present much reduced by the administration of saline and antimonial medicines. The swelling is diminished in the immediate neighbourhood of the seton-threads, but appears more diffused towards the right side of the neck.

12*th*. Tumour growing less; a plentiful and offensive discharge of matter continues. The patient can swallow without difficulty.

22*d*. Bronchocele daily decreasing; the remaining portion feels hard, and the integuments over it are tender. The fetor of the discharge is less.

29*th*. Tumour gradually diminishes; deglutition can be performed without difficulty.

*May* 2*d*. The little patient now left the hospital, with injunctions to attend occasionally. The tumour is not larger than a small chesnut, tender, and hard to the touch. She was ordered to persevere in the use of the seton.

*Remarks*. The result of this case has, I believe, exceeded the operator's expectations; for, taking into consideration the excessive hardness, and the duration of the enlargement, and the failure of the iodine which was so long tried, both internally and externally, he was induced to think that nothing short of extirpation would have any control over the complaint. The event shews that the seton may be introduced, as a last resource and with prospect of success, in the treatment of bronchocele, after other remedies have failed, and that even in the indurated species. The seton has not been frequently employed for bronchocele at this hospital; but I may state that, when used, it has for the most part been attended with success. The last case in which it was tried before the present, was that of a female patient of Mr. Wickham's about three years since, in whom the tumour was of immense size, and had existed for some years, uninfluenced by any means that art could suggest. It was then, unfortunately, of no service. With respect to the powers of iodine, I may here observe, that it is the general opinion at this hospital, that its efficacy is greater when used locally by friction of the unguentum potassæ hydriodatis, than given internally in the form of tincture.

---

VII.

AMPUTATION OF A FINGER WITH ITS METACARPAL BONE, FOLLOWED QUICKLY BY DEATH.

Peter Osman, æt. 50, farming labourer, was received into the hospital, on the 8th of March, under the care of Mr. Lyford, on account of a large warty excrescence on the back of the right hand, situated over the metacarpal bone of the little finger, which was permanently crooked and motionless.

*March* 12*th*. Mr. Lyford to-day removed the tumour, and the little finger, together with its metacarpal bone, at the articulation of the latter with the unciform bone, forming a flap from the inside of the palm. No vessels required ligatures, and the wound was dressed with adhesive plaster and bandage.

13*th*. The patient appears easy, and has slept well; he complains of nothing, having had relief in his bowels twice from castor oil.

14*th*. Has complained some little time of pain in his hand, which has prevented rest. The hand now seems to be in a state of inflammation, which has extended some distance up the fore-arm. There is some degree of excitement, with a flushed face, quick pulse, and hot skin. Ordered liquor. plumb. subacet. dil. to be applied to the fore-arm and hand, and a dose of house mixture statim.

15*th*. Passes his stools involuntarily, and every symptom of debility is developed. There are languor, profuse perspiration, and tendency to sleep, with intolerance of light and sound. Pulse 100, small, and compressible. Thirst, and a foul tongue. R. Conf. opii, ℈j. aquæ carui, ℥j. ♏. statim. capiend.

16*th*. Diarrhœa still continuing, and debility increased—no power even of articulation—no inclination to take food, or observation of things passing around him—slight delirium at times. The wound looks very unhealthy, and the inflammation is extending. Pulse unaltered. R. Opii gr. j. 6tâ quâ horâ sum.

17*th*. Pulse scarcely perceptible, with increased weakness of the patient—there is a constant muttering, as in delirium, and his eye is fixed and glassy—there is a distension of the abdomen, with tenderness on pressure—he lies with his knees raised, and his fæces pass involuntarily from him—little or no urine has been voided—there are occasional eructations —the muscles of the neck and jaw seem stiffened, as nothing can induce him to protrude his tongue—there is likewise some little swelling of the face. The wound is very offensive, and the major part of the upper arm is involved in the inflammation. Cont. Opium. Brandy, ℥ss. o. horâ.

10, P.M. Evidently sinking; subsultus tendinum—great difficulty of breathing, with a rattling noise—the abdomen more tumid. Pulse so irregular as scarcely to be felt. Cont.

18*th*. His strength has gradually declined, and he expired at 5 o'clock this morning—being not quite six days from the time of operation.

On post-mortem examination, nothing was observable, particularly deviating from the healthy structure, with the exception of enormous distension of the stomach and intestines from flatus, especially the larger ones, with some little effusion of serum in the cavity of the peritoneum. The viscera of the chest were healthy. There was some water at the base of the brain, which was attributed to debility, or the last struggles of life.

The above is a melancholy instance of direct constitutional irritation, from a very slight operation.

---

### VIII.

### CASE OF DEEP-SEATED INFLAMMATION OF THE EYE, TREATED BY W. J. WICKHAM, ESQ.

Mary Ray, æt. 21, of corpulent and florid appearance, applied at the hospital, having lost the sight of her right eye. She reports that a slow but continued inflammation has existed in the eye for two years, during which time her vision has gradually become more and more indistinct, till, at last, she is entirely dark. The conjunctiva has no appearance of inflammation, and the sclerotica possesses its usual whiteness. The pupil is immoveably contracted, and a margin of lymph, of a brownish colour, surrounds its edge. She has suffered much from pains of the head, and in the depth of the eye-ball, during the attack, and likewise at the time of her admission. Considering this a case of chronic inflammation of the choroid and iris, and understanding that the usual antiphlogistic treatment had been fully and repeatedly gone through, Mr. Wickham commenced at once with a brisk mercurial action. Calomel, in combination with opium, was given to affect the mouth, which occurred in five days from the commencement. As soon as this effect was sensible and evident, amendment took place in the vision; she became sensible of objects before her eye, and, under the slight continuance of the plan, by degrees recovered her sight completely. Soon after this, the left eye became attacked with a deep-seated inflammation, which was again treated by another quantity of calomel, sufficient to affect the mouth. These attacks, after a time, were again and again renewed, and only yielded to the repetition of a mercurial action.

*April* 30*th*. At the close of an attack of inflammation, after a free use of calomel, Mr. Wickham ordered the Quin. sulph. gr. iij. ter die, in the hopes of altering the disposition to a repetition of the attack. Slight attacks of superficial inflammation occurred subsequently, but, in the course of three weeks, she appeared well. This woman has been troubled with incontinence of urine, which has yielded to the use of the uva ursi in large doses.

*Remarks*. This case shews the effect of mercury in reducing action of the minute vessels of the eye, and aiding the act of absorption; and that, having subdued the chronic affection of one eye, it has been productive of that repeated inflammation of the iris in the other, which is so troublesome and obstinate in ordinary cases of iritis. Here no syphilitic disease is to be traced.

---

### IX.

### CASE OF NASAL POLYPI, CAUSING GREAT DISFIGUREMENT OF THE COUNTENANCE.

On Monday, Feb. 27th, Mr Lyford extracted from the nose of George Smith, fifty-eight years of age, several immense polypi, which had been of four or five years' duration. Both nostrils were distended throughout their whole course by their growth, but more particularly the right one, causing great distortion of the face. The right eye was protruded from its socket slightly forwards, and about an inch and a half outwards, vision being thus impaired; the lachrymal passage was obstructed, forming fistula lachrymalis; the os unguis, as far as could be judged, totally absorbed, and a fetid sanious discharge proceeded from each nostril. The patient complained of constant pain in his head, referred chiefly to the right temple; there were want of appetite, and privation of sleep at night, from the continual pain. He was exceedingly debilitated, and his health was seriously undermined. The polypi were removed by the forceps, but the operation was necessarily tedious, as they extended far upwards. The nose bled freely, and was well cleaned, internally, by cold water, thrown up with a syringe. The parts adjoining the nose were kept wet, after the operation, with a lotion of muriate of ammonia.

*Feb.* 28*th.* The neighbouring parts are swollen considerably, but the forehead is free from pain. There was some bleeding from the nose yesterday evening, which appears to have been rather serviceable than otherwise. A purgative draught was ordered; leeches were applied to the temples, and the lotion was directed to be continued.

During the first few days from this period, the inflammation of the parts was considerable; but was reduced by the application of leeches, bread poultices, and the poppy decoction. The head was not painful, and the patient enjoyed undisturbed sleep at night, which had been unknown to him for months before.

*March* 7*th.* The pain in the right temple has not returned since the polypi have been taken away, and the swollen state of the parts is materially abated; the nose is reduced to one-half its former size; the eye no longer displaced, and its sight good; respiration can be performed through the nostrils without difficulty, and the countenance of the man, however deformed on his admission into hospital, is now natural. The forehead, eyes, and nose, are constantly moistened with the muriate of ammonia lotion.

The man now began to complain of unpleasant sensations at the scrobiculus cordis, want of appetite, and nausea, to relieve which, some tonic and alterative medicines were prescribed. He remained in the house till the third week in March, taking various stomachics, and other remedies for the disorder of the stomach and alimentary canal, when he left by his own desire, the gastric affection being rather aggravated than otherwise by his continuance there, and his spirits broken. But the appearance of the nose and neighbouring parts was quite natural; the swelling had entirely subsided, breathing through the nostrils was free, and the passage for the tears unobstructed.

*Remarks.* The present case I have thought not less worthy of record on account of the magnitude and number of the polypi of the nose, than as demonstrating what displacement parts and organs essential to life will undergo, for a considerable length of time, in consequence of pressure, and how speedily, on the removal of that cause, they will resume their natural situation and powers. In this instance, as above said, the right eye was protruded forwards an inch and a half, and the sight affected; and fistula lachrymalis was produced, by the obstruction of the lachrymal duct; but, soon after the extraction of the nasal polypi was effected, the organ of vision was restored to its proper situation, the imperfection of sight disappeared, and the tears flowed through the right channel.

---

### X.

### FRACTURE OF THE CERVIX SCAPULÆ.

*Case.*—Charles Ballard, æt. 66, stout and muscular, and by trade a blacksmith, was received into the Winchester Hospital, under the care of Mr. Wickham, on the 16th of March, having injured the right shoulder-joint by a fall, whilst walking fast. He stated that the accident happened six weeks before his admission; that he fell with great force on his shoulder, and, immediately afterwards, found he was incapable of moving his arm, and was obliged to support it in a sling, till he reached home. The arm and shoulder swelled a little immediately after the accident.

Upon application for surgical advice, on the following day, he was told that it was a dislocation of the humerus, and the pullies were applied, with the view of reducing the supposed dislocated bone; when the surgeon, after having employed them for some time, imagined that the reduction was effected, and, with this belief, applied a bandage, to support and strengthen the parts injured. After having worn the bandage for three weeks, with the arm supported in a sling, the patient was led to believe that all was right, although he had no motion of the fore-arm or hand; but, on the bandage being removed at the end of that time, the os humeri slipped down into the same position as before, being the cause of great pain. He then applied to several other practitioners in his neighbourhood, none of whom, it appears, could discover the nature of the accident; or, at least, nothing had been done by which the man was relieved, before he was admitted into this hospital, a period (as above stated) of six weeks from the receipt of the injury.

At the time of admission, the shoulder exhibited the appearance of a dislocation of the os humeri into the axilla, so that it might easily have been mistaken for that accident by a superficial observer; the shoulder was sunk, and the head of the humerus could be felt in the axilla; there was constant and severe pain in the joint, except when the arm was kept at rest in one particular position; and the motion of the fore-arm and fingers had been lost ever since the occurrence of the accident. By elevating the humerus, and confining the arm close to the side, thus supporting the glenoid cavity, the joint was free from pain, and of its natural form, whilst, on removing the support, the shoulder dropped, and the uneasy sensations returned; and by placing the hand on the top of the shoulder, with the forefinger fixed on the coracoid process of the scapula, and rotating the humerus, a crepitus was readily perceived: from these diagnostics, the existence of a fracture of the neck of the scapula was no longer doubted. Rollers were applied in the manner of the clavicle bandage, the axilla being supported by pads. The arm was secured close to the ribs by a bandage, to prevent any motion, and the fore-arm was supported in a sling.

*April 26th.* The shoulder-joint has been perfectly easy ever since it has been enveloped by the bandages; and, for the last two or three weeks, the man has daily reported that he has felt the whole extremity acquiring more strength; no pain is felt on pressing near the seat of fracture, and bony union seems to have taken place. The rollers, having become loose, were now re-applied, and passive motion of the limb was allowed to be made gradually.

*May 27th.* Strips of empl. thuris comp. have been applied around the shoulder, and passive motion of the arm has been daily performed. Capability of moving the fore-arm and fingers has gradually increased; he can move the arm in every direction, but cannot yet hold any thing weighty in his right hand. He will leave the hospital in the course of a few days.

No better example than the above can be cited, to verify the remark made by Sir A. Cooper:—" The accident which is much more liable to be mistaken for dislocation, is the fracture through the narrow part of the cervix scapulæ."

---

### XI.

### OPERATION OF LITHOTOMY.

John Gordon, nine years of age, naturally a delicate child, was brought to the Winchester Hospital in the month of February, by his father, a seafaring man, having symptoms of stone in the bladder. He had been lately sounded by a practitioner at Portsmouth, who pronounced it to be a case of calculus. The disease had existed for the last five years; the poor boy was much emaciated, and his sufferings were most acute. On the first introduction of the sound, by Mr. Lyford, (on the day subsequent to admission) the calculus was readily detected. The little boy's bowels were attended to; his mind preserved tranquil and amused by the younger patients of the same ward, with whom he associated, and six minims of solution of potassa, exhibited thrice a-day, which greatly mitigated the severity of the symptoms.

On the 27th of February, the lateral operation, which is the one, I believe, invariably adopted by the surgeons here, was performed, and a calculus, weighing ʒij. ℈ss. extracted from the bladder. The operation occupied rather less than three minutes. The instruments used, were Mr. Key's knife and straight staff, which Mr. Lyford employed in a former case of a little boy, eight years old, in the month of August, 1825, attended with success equal to the present.

The patient bore the operation extremely well; a piece of lint was lightly deposited in the wound, and the knees elevated, and tied together, after he was placed in bed. The urine passed though the wound without impediment; the child's spirits were good, and temper unruffled; not an untoward symptom presented itself during the whole of the after-treatment; the wound granulated favourably, and the water was discharged through the natural outlet on the 13th of March.

The little patient was discharged, cured, on the 26th.

GEORGE BURY.

*Winchester,*
*May 27th,* 1827.

---

II.

*June 5th,* 1827.

SIR,—I request the favour of your inserting the accompanying letters and subjoined observations, in the next Number of the Medico-Chirurgical Review.

I am, Sir, your obedient humble servant,

EDWARD HARRISON.

*Copy of Dr. Harrison's Letter to Dr. Chambers.*

7, *Holles Street, Cavendish Square.*
*May 12th* 1827.

SIR—I was not a little surprized, on my return to Quebeck Street, last Sunday evening, to learn that you had formally refused to meet me in consultation, because I had not received a licence to practise medicine from the London College of Physicians.

As the delicate sufferer was, at the time, in the greatest possible danger, I leave you to form your own conclusions, upon the humanity and propriety of declining to give assistance to an afflicted fellow-creature, in compliance with a capricious and untenable bye-law.

To the patient and to myself the determination was fortunate, because it had led the parents, before my arrival, to procure the assistance of an experienced and able physician. This gentleman has, like myself, thought proper not to apply for the College Licence, and yet he assures me, that the members of your Corporation, do not object to consult with him, whenever their services are wanted; so true it is, that it may suit them at times, to enforce the rigid observance of a byelaw, and at other times to leave it entirely to individual discretion.

To enter into a minute investigation of the supposed grounds of your refusal, would lead me far beyond the limits of an ordinary letter. It will be sufficient for my present purpose to state, that neither the late Dr. Baillie nor your colleagues, Drs. Warren, Turner, or Paris, ever ventured upon such a measure, when their medical services were requested, along with mine. And it would perhaps have been more suitable to a person in your professional station, to have imitated their example, than to have formed a rule for yourself.

As far as concerns me individually, it is really a matter of perfect indifference, whether I am in future to meet in consultation with the Fellows of your College, or am to lose their services in cases of danger, or obscurity. London, happily, contains many physicians, out of the pale of your corporation, in whose skill invalids may safely confide.

Under this impression, my first determination was, wholly to overlook the contents of your note addressed to the mother of my patient; but, on referring to the purport of it, a few nights since, in a large party of physicians, who, in the phraseology of your College, are denominated "*alieni homines*," I became convinced of my error. Indeed it now appears to me, that, in following the bent of my inclination, I should have neglected the duty I owe, to my alma mater, the University of Edinburgh,—to my brethren the "alien," Physicians established throughout the British Dominions,—and to the public at large.

Deeply interested in the questions at issue between the Medical Graduates of England, and of all other countries, I shall now call your attention to some of the reasons, which have led me uniformly to resist the arrogated powers of the London College. In opposing them, I am neither influenced by hostility nor prejudice. My chief aim is to relieve myself and brethren from the degradations imposed upon us.

Among the reasons which have influenced me to adopt my present course, it will be sufficient to state, 1st, That, unless I have been misinformed, every candidate for your Licence is obliged, on his bended knees, to swear obedience to the laws and regulations of the College, though, by a refinement in legislation, as far as I know, peculiar to yourselves, he is not suffered to read them either before or after he has complied with the oath! If such be the case, I cannot help giving it, as my deliberate opinion, that the ceremony is equally dishonorable to the parties who require, and to those who submit to this preposterous exhibition.

*2ndly*, Another insuperable objection to the College Licence is founded on your arbitrary and illegal Bye-laws. According to my interpretation of the medical statutes, the College of Physicians is equally open to the Graduates of every university. It possesses no distinction of rank, though the highest has, by a series of encroachments, been limited to the Physicians of Oxford and Cambridge, while a lower grade has been forced upon all other Physicians.

These are some of the numerous objections which I feel, and which make it impossible for me, under the present constitution of the College, to apply for their licence. Should the College still be of opinion, as they formerly professed to maintain, that they can legally compel the acceptance of a licence, or the discontinuance of practice, I beg them to be assured, that I am perfectly ready to try the question, whenever they may think proper to afford me the opportunity. I must, however, in the mean time, strongly remonstrate against the custom of endeavouring to obtain their object by a course injurious to medical science, and prejudicial to the community.

You may possibly be aware, that I formerly stated the same sentiments to Dr. Baillie; and, after his death, to Dr. Turner. I did not omit, on either occasion, to add, that the Fellows were, in my opinion, highly culpable in making regulations, which they dare not attempt to enforce in a Court of Law.

As my sentiments remain unaltered, I embrace the opportunity which you have afforded me, to renew my offer through you to the College. Should the challenge be at length accepted, I pledge myself to carry the suit to a full hearing and final decision.

In repeating my offer for the third time, I desire to remind you, that I have hitherto been content to assert my own privileges and independence, when they were unnecessarily assailed. But after so many provocations I now think myself called upon openly to claim for myself and colleagues, all the rights and privileges of British subjects, agreeably to the union of the two kingdoms. To an Englishman, it appears to be more than absurd, and ridiculous, that he should be supposed to have lost any of his natural rights, by visiting another portion of the same kingdom, merely to qualify himself for the duties of a profession, the knowledge of which he could no where acquire, in his own part of the country.

If the Fellows shall still think fit to decline the contest, an enlightened public cannot fail to appreciate their real motives, however they may be disguised or concealed.

As for the Graduates of my order, they will not be slow to perceive the folly of connecting themselves with a Corporation from which they must afterwards expect to receive only marks of neglect, of opposition, or of humiliation.

I think myself entitled explicitly to enquire from you, on this occasion, whether, in refusing to meet me in consultation, you considered yourself as acting discretionally, or under an indispensable obligation imposed on you by the byelaws of the College.

I beg leave to add, in conclusion, that, unless I receive a satifactory answer, in the space of a month, either from you or the College, to the several allegations contained in this Letter, I shall feel it my duty to publish it, for the information and guidance of my brethren, wherever they may be situated.

I am, Sir, your obedient humble servant,

(Signed) EDWARD HARRISON.

*To Dr. Chambers, Brook Street.*

---

*Copy of Dr. Chambers' Answer to Dr. Harrison's Letter.*

*Brook Street*, *May* 14*th*, 1827.

Sir,—I have to acknowledge the receipt of your letter, dated the 12th of May, which only reached me this afternoon.

In answer to it, I beg leave to state that I do not feel myself called upon to enter into the discussion of the questions which you conceive to be at issue between the College of Physicians and yourself.

I have only to say, as to myself, that, in refusing to meet you in consultation, I acted in obedience to a positive regulation of the College, and that it is a matter of indifference to me whether you publish your letter on the subject or not.

I am, Sir, your most obedient,
Humble servant,
(Signed) W. F. CHAMBERS.
To Dr. Harrison.

REMARKS.

1*st*. Although Dr. Chambers declares that, in refusing to meet me in consultation, he acted in obedience to "a positive regulation of the College," will he venture to maintain that he has never invaded this *positive regulation* or bye-law, during his connexion with that body? I have, as already observed, been joined in practice with no fewer than four Fellows, during my short residence in London;—I may now add that, from Dr. Chambers alone, have I encountered a refusal. I have also said that the *alien* physician, an old metropolitan practitioner, who supplied the Doctor's place, is in the constant habit of meeting the Fellows professionally. After stating these facts, I shall not expatiate further upon the glaring incongruities and absurdities of the Fellows, but leave them to explain their motives, and to form their own justification.

2*dly*. Do the Fellows ever decline to consult with surgeons on cases strictly medical? Physicians had formerly the whole management of constitutional diseases entrusted to them, and were also applied to as the dernier resort in surgery; but so completely are the tables now turned in these respects, that while the surgeon openly beards the Doctor, in medical practice, he is jealous of the smallest encroachment upon his own department. Many examples of recent date might be given in support of these assertions. As regards the former, the reader cannot have forgotten, that two individuals of the highest medical and surgical rank, were lately in conjoint attendance, for several successive weeks, upon two distinguished and very exalted characters. One of the cases was *purely medical;* and the surgical treatment of the other was so inconsiderable, that the surgeon could only be wanted for his medical skill.

3*dly*. According to present usage in this country, the ordinary practice of physic is almost entirely confided, in the first instance, to the family apothecary. The physician is only thought of when the case becomes alarming or tedious. After his introduction, their visits are continued in accordance, and the two share the responsibility between them.

4*thly*. Upon what justifiable grounds, then, can the Fellows refuse to be united in consultation with the "independent physician," whilst they have no hesitation in freely consulting with the surgeon and apothecary?

5*thly*. In a colloquial conversation with the late Dr. Baillie, so long ago as the month of June, 1821, while we were engaged in the case of a young lady, I fully explained my opinion of the London College of Physicians as alluded to in my letter to Dr. Chambers. This was the third patient, after my arrival in London, who had called for our joint assistance. As the Doctor had never omitted, on former occasions, to recommend my application for the College Licence, I determined, at this interview, if a good opening occurred, to assign my reasons for declining to comply with his urgent solicitations. The opportunity being given, I avowed it as my deliberate conviction, arising from legal inquiries, and a careful investigation of the subject,—

"1*st*. That the College of Physicians is, according to the laws of the Realm, and charter of King Henry VIII. equally open to the medical graduates of every university. I added that it was, in point of fact, conducted upon this principle, from the first establishment in 1523, to about the middle of the last century, including a period of more than two hundred years.

"2*dly*. That at this eventful era, a predominant party of Oxford and Cambridge Physicians, unfortunately for medical science and the true interests of their profession, had the temerity to narrow the Bye-laws, in order to promote their own selfish views. In referring to the exact time when these regulations were enacted, we are led to believe that they were chiefly intended to check the rising prosperity of the University of Edinburgh. Had the College formed their excluding Bye-laws anterior to the British Union, something might perhaps have been advanced in extenuation of their conduct, though, inasmuch as the healing art is the production of no particular soil, it would be absurd to attempt to confine its cultivation within the limits of any district. But, no sooner were the two nations consolidated into one kingdom, than it became the bounden duty of every citizen to efface local distinctions, and promote harmony through the land.

" *3dly* That the College was extremely culpable in making Bye-laws, which they durst not endeavour legally to enforce.

" *4thly*. That it was due to themselves and to the physicians of my order, either to try the validity of their present regulations, or to make such as they would be able to defend.

" *5thly*. That, fully satisfied with the stability of my own position, I was ready, whenever the College were pleased to attack my station, to defend it with legal and constitutional weapons."

Such was the purport of my conference with Dr. Baillie, at our last interview, and a similar, though less extended conversation, took place in the year 1824, between Dr. Turner and myself. Having subsequently been met in consultation, both by Dr. Paris and Dr. Warren, judge of my surprize, on receiving a positive refusal, in the person of Dr. Chambers.

